# Evaluating Georgia’s Cystic Fibrosis Newborn Screening Algorithm to Inform Improvement Recommendations

**DOI:** 10.3390/ijns11040087

**Published:** 2025-09-29

**Authors:** Brittany Truitt, Eileen Barr, Angela Wittenauer, Andrew Jergel, Shasha Bai, Rossana Sanchez Russo, Kathryn E. Oliver, Kathleen McKie, Rachel W. Linnemann

**Affiliations:** 1 Department of Pediatrics, Emory University, Atlanta, GA 30322, USA; brittany.ashton.truitt@emory.edu (B.T.); andrew.jergel@emory.edu (A.J.); shasha.bai@emory.edu (S.B.); kolive3@emory.edu (K.E.O.); 2 Children’s Healthcare of Atlanta, Atlanta, GA 30329, USA; 3 Department of Human Genetics, Emory University, Atlanta, GA 30322, USA; alwitte@emory.edu (A.W.); rossana.sanchez@emory.edu (R.S.R.); 4 Department of Pediatrics, Augusta University, Augusta, GA 30912, USA; ktmckie@augusta.edu

**Keywords:** cystic fibrosis, newborn screening, case detection, health equity, delayed diagnosis

## Abstract

Early diagnosis by newborn screening (NBS) has contributed to improved outcomes in children with cystic fibrosis (CwCF). Georgia’s two-tiered algorithm consists of a fixed immunoreactive trypsinogen (IRT) cut-off followed by a 39-variant *CFTR* genetic panel. We conducted a retrospective review of CwCF born in Georgia from 2007 to 2022 to evaluate false negative NBS frequency. We characterized CwCF whose diagnosis was delayed beyond 28 days of age despite positive NBS. Six cases were detailed demonstrating the impact of missed and delayed diagnoses. We examined IRT trends from 2018 to 2022 and cut-off approaches. Missed case detection by expanded *CFTR* variant assays was assessed. Of 390 CwCF born in Georgia, 18 (4.6%) had false negative NBS—6 due to lack of *CFTR* variant detection and 12 due to low IRT values. Thirty children had delayed diagnosis, with the majority related to sweat testing. Minoritized children made up 19% of the population but 43% of missed and 44% of delayed diagnoses. Black and Hispanic infants had higher odds of missed or delayed diagnosis compared to non-Hispanic White infants (OR = 2.7, *p* = 0.027 and OR = 6.1, *p* < 0.001, respectively). Average IRT values varied across kits and were lower in warmer seasons. Expanded *CFTR* assays would reduce missed cases. Our results informed recommendations for improvement at multiple steps in the NBS process.

## 1. Introduction

**Figure 1:**
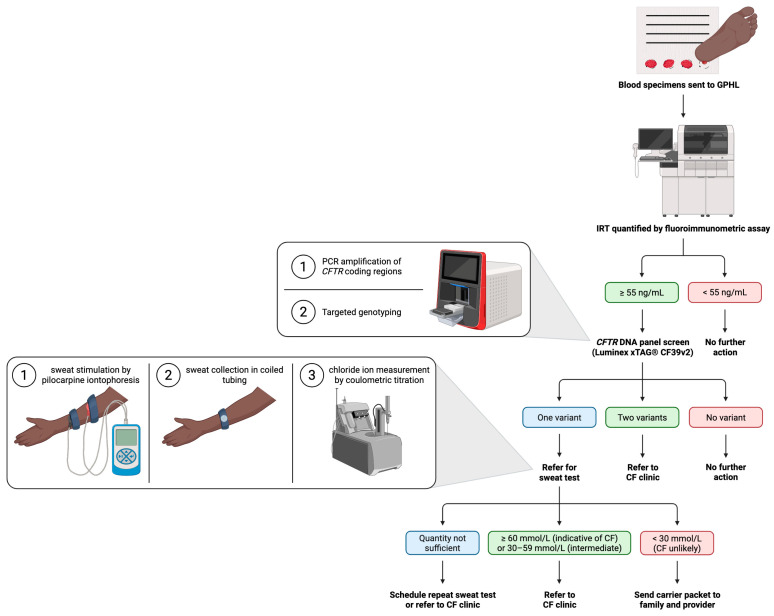
The CF newborn screening algorithm in Georgia. Dried blood spots are collected by a heel prick between 24 and 48 h of age and sent to the Georgia Public Health Laboratory (GPHL). Immunoreactive trypsinogen (IRT) concentration is quantified by a Genetic Screening Processor (GSP) Neonatal IRT kit that employs fluorescence-based detection of a specific antigen on the IRT molecule. If IRT levels are ≥55 ng/mL, the sample is screened for the presence of 39 *CFTR* variants included on the Luminex xTAG^®^ CF39v2 panel. This method works by amplifying *CFTR* coding regions via polymerase chain reaction (PCR), followed by targeted genotyping. If no variants are detected, the NBS is reported as negative. Georgia does not employ a very high IRT cut-off. If one or more *CFTR* variants are detected, the NBS is considered positive, and the case is sent to the state’s NBS Follow-up Program for further evaluation. If two *CFTR* variants are detected, the patient is referred to a CF clinic for evaluation and sweat chloride testing. Should only one *CFTR* variant be detected, the patient is referred for a sweat chloride test by their primary care provider. The technique employs pilocarpine iontophoresis to activate production of sweat, which is collected in coiled tubing and subjected to coulometric titration to measure chloride ion concentration. Patients with sweat chloride values indicative of CF, or within the intermediate range, are referred to a CF clinic. Results returned as “quantity not sufficient”, which occurs when an insufficient amount of sweat is collected, trigger repeat sweat testing or referral to a CF clinic.

Since the implementation of universal newborn screening (NBS) for cystic fibrosis (CF) in the United States (U.S.) in 2010, most infants are now diagnosed with CF by 60 days of age [1]. Timely diagnosis allows for early intervention which has reduced malnutrition, led to more rapid increase in lung function, and increased survival [2,3,4]. Currently, there is significant variability in the screening algorithms employed by each state. NBS for CF began in Georgia in 2007. Georgia uses a CF NBS algorithm that first measures immunoreactive trypsinogen (IRT) concentrations in dried blood spots from newborns, with values ≥ 55 ng/mL triggering Cystic Fibrosis Transmembrane Conductance Regulator (*CFTR*) genetic testing. This type of fixed IRT cut-off approach is used in approximately half of U.S. states, while other states employ a floating IRT cut-off in which values above the daily 95th–98th percentile undergo second-tier genetic testing [5]. Georgia uses a 39-variant *CFTR* DNA panel (Luminex^®^) as its second-tier test, which demonstrates lower sensitivity among minoritized populations [6]. Infants with two *CFTR* variants detected on NBS are referred by their primary care provider directly to a CF care center in Georgia, while infants with only one variant detected are referred by their primary care provider for sweat chloride testing. Infants with an elevated IRT, but no *CFTR* variants detected, are considered to have a negative NBS despite the high IRT level (Figure 1). Positive screens are sent from the state lab to the state’s NBS Follow-up Program, which coordinates follow-up for the infants until a final outcome is determined (Figure S1).

The Cystic Fibrosis Foundation recommends that infants identified by NBS should complete sweat testing and obtain a diagnosis by 4 weeks of age [7]. Median time to first CF care event has decreased over time as NBS protocols are refined and improved across states [8]. However, registry data have also shown that minoritized infants are diagnosed later than non-Hispanic White infants with CF and have poorer early nutritional outcomes [9,10]. Minoritized people with CF (PwCF) have increased morbidity and mortality compared to non-Hispanic White PwCF and are less likely to be eligible for CFTR modulator therapies [11,12]. Thus, early and equitable diagnosis is key to reducing health disparities.

The primary aim of this study was to evaluate Georgia’s NBS algorithm by identifying children with CF (CwCF) born in Georgia who were diagnosed after a false negative NBS or after 28 days of age despite a positive NBS and analyze outcomes by race and ethnicity. Our secondary aim was to examine IRT trends in Georgia including seasonal and kit-related variation to evaluate performance of various IRT cut-off approaches. Data from this study would be used to inform improvement recommendations for CF NBS in Georgia.

## 2. Materials and Methods

### 2.1. Study Design

This statewide study of Georgia’s NBS algorithm was conducted via a collaboration between the Children’s Healthcare of Atlanta and Emory University CF Care Center and the Augusta University CF Care Center (the two CF Foundation accredited care centers in Georgia), as well as the Georgia Department of Public Health (DPH). This study was approved by the Institutional Review Boards at Emory University (#00005155), Augusta University (#1980623-4), and the Georgia DPH (#230104).

A retrospective review of CwCF born in Georgia from 2007 to 2022 was performed to identify cases missed by NBS. NBS data were obtained from the state Newborn Screening Follow-up Program in partnership with the Georgia DPH and the Georgia Public Health Laboratory (GPHL). Cases missed by NBS, meaning those with a falsely negative NBS, were detected using CF Foundation Patient Registry (CFFPR) [13] data, queries of CF clinic providers, and review of electronic medical records (EMR). Date of CF diagnosis was collected from the CFFPR. For patients with incomplete data in CFFPR, not enrolled in CFFPR, or with diagnosis after 28 days of age, date of CF diagnosis was determined via EMR review based on either date of genetic confirmation, date of clinician diagnosis, or date of confirmatory sweat test, whichever was first. Additional endpoints collected were missed cases due to IRT below the fixed cut-off and missed cases due to no detected *CFTR* variants despite elevated IRT.

CwCF born in Georgia from 2011 (after the state’s most recent IRT cut-off change in December 2010) through 2022 were also evaluated to collect cases of delayed diagnosis > 28 days after birth despite positive NBS. Demographic and clinical data for these infants, including reasons for delayed diagnosis, were obtained using the CFFPR, EMR, and the state’s NBS Follow-up Program database. CwCF with missed or delayed diagnosis who were not born in Georgia and therefore did not have a Georgia NBS were excluded. Median age at diagnosis was calculated for all groups. Sex, race, and ethnicity data for the pediatric CF population born in Georgia were compared to that of those with missed or delayed diagnosis. Illustrative cases were chosen to highlight the impact of a delayed or missed CF diagnosis on clinical presentation.

Endpoints were analyzed according to race and ethnicity using census categories as defined by the standards on race and ethnicity from the U.S. Office of Management and Budget (OMB) [14]. Race was collected from Georgia’s NBS Follow-up Program database, as completed by hospital staff on the NBS card as American Indian/Alaskan Native, Asian, Black, Multiracial, Other, Pacific Islander/Native Hawaiian, Unknown, and White. Ethnicity was documented as Hispanic or non-Hispanic. Missing race/ethnicity data were obtained from the EMRs. American Indian/Alaskan Native, Asian, Multiracial, Other race, and Pacific Islander/Native Hawaiian were combined for some analyses due to small sample size.

IRT values and reagent kit numbers from all NBS performed in Georgia over 5 years from 2018 to 2022 were obtained from GPHL. Floating IRT percentiles were calculated using the method described in the Clinical and Laboratory Standards Institute’s Newborn Screening for Cystic Fibrosis Appendix A [15]. All IRT percentiles were calculated by removing outliers ≥ 170 ng/mL. The percentage of individuals requiring second-tier testing was calculated for multiple IRT cut-off approaches, including fixed, floating, and hybrid (a floating cut-off with a back-up fixed cut-off, should the floating cut-off be unusually high). Analysis was then performed to assess variation in IRT values by season and reagent kit. For seasonal analysis of IRT trends, seasons were defined as spring (March–May), summer (June–August), fall (September–November), and winter (December–February).

Lastly, we examined performance of alternative *CFTR* variant assays at identifying the CwCF missed by NBS, including the Illumina 139 variant assay [16] and two custom assays using disease-causing variants listed in the Clinical and Functional Translation of CFTR (CFTR2) database [17]: (1) a 689-variant panel currently in use in Wisconsin [18] and personal communication, Mei Baker, 2024 and (2) a hypothetical next generation sequencing (NGS) panel customized to identify all disease-causing variants listed in the CFTR2 database as of the most recent 2024 update (1085 variants) that are routinely detectable by NGS in NBS laboratories.

### 2.2. Statistical Methods

Continuous variables are described using medians and interquartile ranges (IQRs) while categorical variables are presented as counts and percentages. Overall group comparisons for race, ethnicity, and missed/delayed CF cases for days to diagnosis used the Wilcoxon rank sum test and Kruskal–Wallis rank sum test (for more than 2 groups). Standardized odd ratios (OR) with 95% confidence intervals (95% CI) were calculated from univariable logistic regression to interpret the relationship between race/ethnicity and missed/delayed CF cases born since 2011 (when delayed case data became available). A sensitivity analysis was also performed, including those born before 2011. A *p*-value less than 0.05 was considered statistically significant. The average IRT concentration and 95th and 96th percentiles trends were graphically provided for the summer and winter seasons and the different kits used from 2018 to 2022. The number of individuals above the fixed IRT value of 55, trimmed 95th and 96th percentiles, and the trimmed percentiles with back-up fixed IRT cut-offs of 55 ng/mL and 60 ng/mL were examined. All data cleaning, statistical testing, and IRT trend graphics were created using R Statistical Software (v4.2.1; R Core Team 2022).

## 3. Results

### 3.1. Evaluation of Missed and Delayed CF Diagnoses

**Figure 2:**
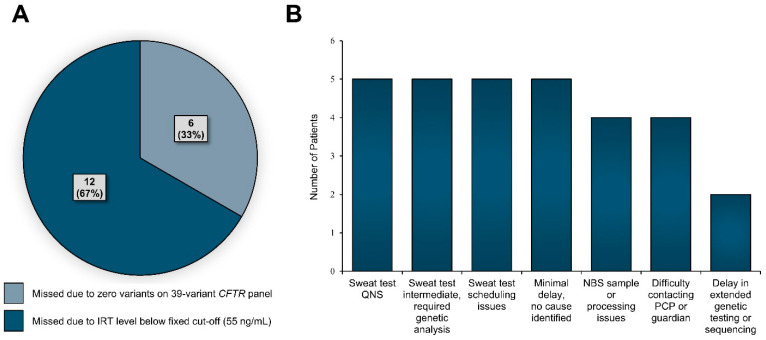
Reasons for missed or delayed CF diagnosis. (**A**) Children with CF born in Georgia from 2007 to 2022 with a missed newborn screening (NBS) diagnosis (*n* = 18). (**B**) Children with CF born in Georgia from 2011 to 2022 with diagnosis at >28 days of age (*n* = 30). Reasons for delay were ascertained from the electronic medical records and NBS Follow-up Program notes. Sweat test QNS (quantity not sufficient) refers to a sweat test with inadequate sweat collection. Sweat test intermediate refers to a sweat chloride ≥30 mmol/L but <60 mmol/L). Sweat testing scheduling difficulties include all aspects of delays in scheduling such as issues with the primary care provider (PCP) ordering the sweat test or not conveying the urgency to guardians and challenges with sweat test scheduling by guardians. Difficulty contacting PCP or guardian signifies that the state Newborn Screen Follow-up Program was unable to make timely contact to report abnormal NBS results to PCP or PCP had difficulty giving results to family. NBS sample quality issues signifies that the initial heel-stick sample was not sent appropriately or unable to be processed, or otherwise not obtained. Minimal delays without significant cause identified refers to those whose NBS resulted appropriately however first clinic appointment for confirmation of diagnosis by provider and/or sweat testing did not occur within 28 days and chart review revealed no clear etiology for the delay. Delay in genetics refers to infants who could not undergo timely sweat testing due to being in a neonatal intensive care unit and had a delay in return of genetic testing results to confirm CF.

From 2007 to 2022, 372 infants were born in Georgia and diagnosed with CF after a positive NBS. We identified an additional 18 missed cases of CF after a falsely negative NBS during that period, giving a false negative rate of 4.6%. Of these, 12 (67%, or 3.1% of total CwCF born in Georgia) were missed due to having an IRT below the fixed cut-off, while 6 (33%, or 1.5% of total CwCF born in Georgia) were missed due to not carrying any genetic variants tested on the state’s *CFTR* panel (Table 1, Figure 2A). Clinical data for missed cases are summarized in Table S1. Of the 12 infants missed due to IRT below the cut-off, 4 (33%) were diagnosed with meconium ileus or plugs. None were born to mothers taking CFTR modulator therapy. The median age at diagnosis for CwCF missed by NBS was 300 days (IQR 88, 1342) (Table S2).

Between 2011 and 2022, 30 CwCF were identified as having a delayed diagnosis > 28 days of age despite positive NBS. Difficulty with sweat testing was the most common reason for delay in 50% of cases, including inconclusive sweat testing and challenges with test scheduling (Figure 2B). Among CwCF with delayed diagnosis, median age at diagnosis was significantly higher for CwCF with only one variant detected on NBS (62 days; IQR 30, 101) compared to those with two variants detected (34 days; IQR 31, 39) (*p* = 0.012, Table S2).

**Figure 3:**
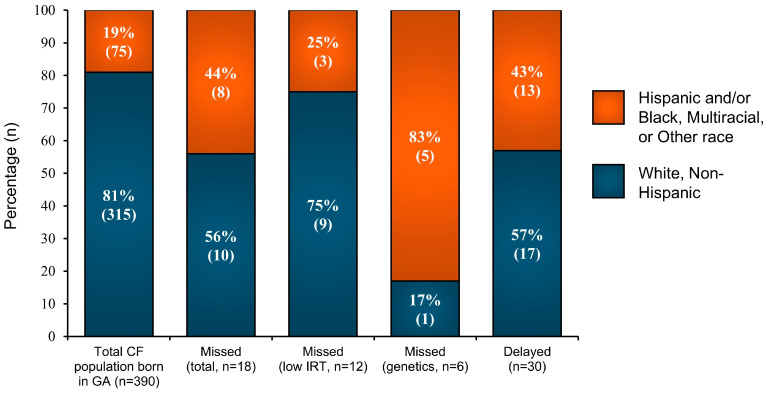
Comparison of missed (*n* = 18) and delayed (*n* = 30) diagnoses in the pediatric CF population born in Georgia between 2007 and 2022 by ancestry group (*n* = 390). Study Periods: Missed Cases 2007–2022; Delayed Cases 2011–2022.

Missed and delayed cases were stratified by race and ethnicity, and this stratification was compared to the total population of CwCF born in Georgia from 2007 to 2022 (*n* = 390) (Figure 3). Notably, minoritized CwCF (identifying as Black, Multiracial, or Other race or Hispanic ethnicity) made up 19% of the pediatric CF population born in Georgia but 43% of the 18 missed cases and 44% of the 30 delayed diagnoses. Of the six cases missed by NBS due to lack of *CFTR* variants identified by the NBS genetic panel, five (83%) were Black, Multiracial, Other race or Hispanic children. Four (67%) had Hispanic ethnicity. Of the missed cases due to low IRT, three (25%) occurred in minoritized children. Univariable logistic regression, using only data since 2011, when delayed case data became available, revealed that Black infants had 2.7 times the odds of a missed or delayed CF diagnosis compared to White infants with CF (95% CI 1.1, 6.2; *p* = 0.027; Table 2). Hispanic infants had 6.1 times the odds of a missed or delayed CF diagnosis (95% CI 2.4, 16; *p* < 0.001; Table 2). A sensitivity analysis including all data since 2007, which assumes all non-missed infants had on-time diagnosis, was consistent with the primary analysis (Table S3). Among those with missed and delayed diagnosis, median age at diagnosis was also numerically longer for minoritized compared to non-Hispanic White CwCF, though these differences were not statistically significant (Table S2).

### 3.2. Illustrative Cases Missed by CF NBS

Case 1: A 20-month-old non-Hispanic White female with history of asthma and recurrent otitis media was evaluated by a pulmonologist for chronic cough and found to have two pathogenic *CFTR* variants on a primary ciliary dyskinesia panel.

This child had a normal NBS (IRT 44.5 ng/mL). She had an unremarkable neonatal course; however, she began to have chronic cough and recurrent respiratory and ear infections around 11 months of age and was diagnosed with asthma. She had adequate growth and no signs of malabsorption. At age 20 months, she was diagnosed with CF after genetic testing demonstrated two disease-causing *CFTR* variants (F508del and 3120G->A) and sweat chloride concentration was 74 and 76 mmol/L (levels ≥ 60 mmol/L are consistent with CF). She was also diagnosed with exocrine pancreatic insufficiency (fecal elastase < 50 µg/g). Chest computed tomography (CT) was completed with evidence of bilateral airspace opacities and mild bronchiectasis.

Case 2: A 6-year-old Hispanic White female with malabsorption symptoms and a sibling with CF was diagnosed with CF after a positive sweat test.

This child had an NBS with an elevated IRT (246.4 ng/mL) and no *CFTR* variants. Per state protocol, this result was considered a negative screen so was not sent to the Follow-up Program. Although the infant had an older sibling with CF diagnosed clinically who had not had a newborn screen, a sweat test was not obtained for this infant due to lack of symptoms. At the age of five she developed symptoms of malabsorption and underwent sweat chloride testing which was positive (100 and 101 mmol/L). She was diagnosed with exocrine pancreatic insufficiency (fecal elastase < 15 µg/g). *CFTR* sequencing showed two pathogenic variants (1811 + 1634A->G and 3271delGG). Chest imaging at diagnosis revealed evidence of mucus plugging, peribronchial thickening, and bronchiectasis.

Case 3: A 4-year-old non-Hispanic Black female with history of meconium ileus and failure to thrive presented to gastroenterology clinic for abnormal stools and was noted to have digital clubbing.

This child was born at 37 weeks gestational age and required exploratory laparotomy with small bowel resection for meconium plugs. Her initial NBS was notable for elevated IRT (416.6 ng/mL) but no *CFTR* variants were detected—considered a negative screen. A CF common variant panel was sent by the neonatal intensive care unit and was negative. She then presented to gastroenterology clinic at age four with failure to thrive and malabsorption symptoms. Sweat chloride was 103 and 105 mmol/L and stool studies revealed pancreatic insufficiency (fecal elastase < 50 µg/g). *CFTR* sequencing showed two pathogenic variants: CFTRdele1 and CFTRdele21. At diagnosis she had a BMI < 5th percentile, *Pseudomonas aeruginosa* growth on cystic culture, and findings of lung disease including consolidation, mucus impaction, peribronchial thickening, and hyperinflation on chest radiography.

### 3.3. Illustrative Cases with Delayed CF Diagnosis Despite Positive NBS

Case 1: A Hispanic infant with positive NBS and failure to thrive diagnosed with CF at 3 months old by sweat testing.

This 3-month-old infant with a positive NBS (IRT 176.7 ng/mL, single variant (3876delA) presented to CF clinic after a positive sweat chloride test (104 and 116 mmol/L). The patient’s mother was monolingual in Spanish and cited difficulties with scheduling the sweat test. At presentation the infant had severe malnutrition and weight < 0.01 percentile which required hospital admission. *CFTR* sequencing revealed a rare second pathogenic variant c.1811del (no legacy name) not listed in CFTR2. During admission she also had anemia and vitamin K deficiency and required supplemental oxygen and antibiotics for pulmonary exacerbation. Due to malnutrition, she requires a feeding tube.

Case 2: A 5-week-old Hispanic male infant with positive NBS admitted to the hospital for failure to thrive and subsequently diagnosed with CF.

This infant’s NBS was positive with elevated IRT (172.1 ng/mL) and 1 variant (F508del). After numerous visits to the pediatrician for poor weight gain and reflux, this infant was admitted for failure to thrive with weight at the 2nd percentile. Sweat testing during admission was positive (sweat chloride 90 mmol/L). The patient’s mother reported that the primary care provider did not emphasize the urgency of the sweat test, despite the infant’s symptoms and NBS result, because of decreased suspicion of CF due to the patient’s ethnicity. *CFTR* sequencing revealed a second variant (S466X).

Case 3: A 3-month-old non-Hispanic White infant with a positive NBS presented to the emergency department with edema and hypoalbuminemia.

This infant had a positive NBS with elevated IRT (120.4 ng/mL) and one pathogenic variant F508del. He had initial sweat testing around 12 weeks of age with a quantity not sufficient collection. Several weeks later, he was admitted for management of hypoalbuminemia (1.1 g/dL) and failure to thrive (<1st percentile). He was diagnosed with exocrine pancreatic insufficiency (fecal elastase < 10 µg/g). *CFTR* sequencing showed a second variant 622-1G->C. Repeat sweat testing was positive (89 and 91 mmol/L).

### 3.4. IRT Trends

**Figure 4:**
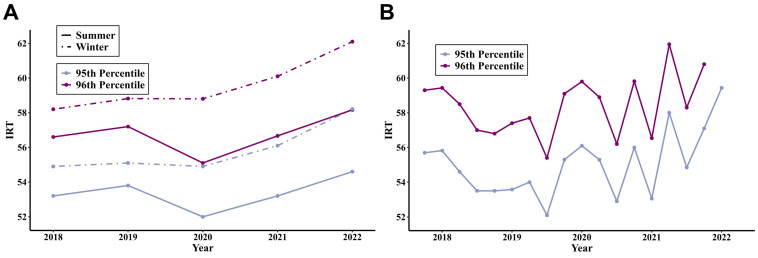
IRT trends of all Georgia newborn screens from 2018 to 2022. (**A**) Seasonal variation in average 95th and 96th daily percentiles calculated for each year. (**B**) Variation in 95th and 96th percentiles by IRT reagent kit (each dot represents a unique kit).

IRT values from all Georgia NBS performed over 5 years from 2018 to 2022 (*n* = 715,639) were analyzed to calculate daily IRT averages and historic daily 95th and 96th percentile cut-offs (Table S4). Yearly mean IRT levels along with calculated daily 95th and 96th percentiles were up-trending in 2021 and 2022 compared to prior years. Seasonal analysis of IRT trends showed lower mean daily floating cut-off values in the summer months compared to winter months each year (Figure 4A). IRT values also showed significant variation among reagent kits (Figure 4B).

### 3.5. Performance of IRT Cut-Off Approaches and CFTR Panels

Using the percentile cut-offs calculated from historic IRT values (2018–2022), known missed cases of CF due to low IRT on NBS were evaluated to determine if any would have screened positive using a floating IRT cut-off approach. Of the four missed cases due to low IRT during this period, none would have screened positive with the use of a floating IRT cut-off at either the 95th or 96th daily percentile. Of known CwCF born in Georgia from 2018 to 2022 (*n* = 99), one child with an IRT of 63.5 ng/mL would have been missed with use of the daily 96th percentile cut-off. This infant would have been detected using the daily 95th percentile IRT cut-off. The hybrid approach with a floating 96th percentile and a back-up fixed cut-off of 60 ng/mL appeared to provide the lowest risk of missed cases while also controlling the number of specimens requiring second tier genetic testing (6929), compared to 8352 with the existing fixed cut-off in 2022 (Table S4).

Georgia’s current 39-variant *CFTR* panel resulted in six children with CF being missed due to lack of variants detected. The Illumina 139-variant assay would have detected at least one variant for four of these six infants (67%) (Table S5). The Wisconsin CFTR2-based panel of 689 pathogenic variants would have detected five of six cases (83%). Of these, one child would have had only a single variant detected. A hypothetical next generation sequencing panel customized to identify all 1085 disease-causing variants listed in CFTR2 as of 2024 that are routinely detectable by NGS in NBS laboratories would still only detect five of six missed cases.

## 4. Discussion

This retrospective review of statewide diagnostic and demographic data for CwCF born in Georgia since initiation of CF NBS demonstrates that minoritized infants were significantly more likely to have a missed or delayed CF diagnosis compared to non-Hispanic White infants. The odds of missed or delayed diagnosis were highest for Hispanic infants. Most false negative NBS were due to IRT below the fixed cut-off, an important finding because expanded genetic testing will not catch these infants. Falsely negative NBS also occurred due to lack of *CFTR* variant detection; for our CF patients this occurred in Black or Hispanic CwCF in five out of six known cases. This finding is consistent with prior reports that the Luminex-39 variant panel has lower case detection among minoritized PwCF [19]. Notably, one of our missed cases related to *CFTR* variant testing with two large deletions would still not have been identified using a panel of 1085 known CF-causing variants from CFTR2 that are routinely detectable by NGS in NBS laboratories, as recommended by newly published consensus guidelines [20].

Previous reports have suggested the sensitivity of CF NBS protocols in the US to be around 90% [19]. The CFFPR estimates the occurrence of false negative NBS from 2011 to 2020 at 3.8% of patients. However, this is likely an underestimate as one third of patients were missing data for this question [19]. Our data revealed that 4.6% of PwCF born in Georgia from 2007 to 2022 had a false negative NBS. Our false negative NBS rate is lower than that found in investigations in both California (9.2%) and Colorado (10.3%), likely owing to our relatively lower IRT threshold compared to California and the IRT/IRT/DNA approach in use in Colorado [21,22]. Interestingly, more false negative NBS in California were due to incomplete *CFTR* variant detection, though this proportion was lower in Hispanic infants, reflecting California’s intentional addition of *CFTR* variants commonly found in Hispanic CF patients to their NBS algorithm [21].

CFFPR data showed that Asian, American Indian, Black, Hawaiian/Pacific Islander, Two or more race, and Hispanic CwCF were over-represented in the category of having a false negative NBS (29.9%) when compared to their overall proportion in the CF population (15.4% based on 2020 data) [19]. Our data aligns with these findings and reveals an even more striking conclusion, with minoritized individuals making up 44% of our known cases missed by NBS. Though the false negative CF NBS rate in Georgia appears lower than some other states, the large inequity in prompt diagnosis for infants of all races and ethnicities remains and is vital to address. Even among the group of children with missed and delayed diagnosis, time to diagnosis was longer for minoritized CwCF, suggesting that provider biases and other barriers to testing may contribute to delays in diagnosis.

The clinical impact of having a false negative NBS for CF, and thus a late diagnosis, has been described in case reports revealing manifestations of severe lung disease and malnutrition [23]. This has prompted many states to evaluate their own screening algorithms and often resulted in lowering of IRT thresholds and/or expansion of *CFTR* variant panels [21,22,23,24]. To demonstrate the impacts of delayed and missed diagnoses and highlight some of the challenges observed with our current screening algorithm, we describe in detail three missed and delayed cases. Of note, several of our missed cases had siblings diagnosed with CF. Some parents decline sweat testing of a healthy-appearing sibling with a normal CF NBS, yet our findings reiterate the importance of obtaining sweat testing for all siblings, even in the era of NBS.

Meconium ileus has a known association with lower IRT values which can result in a falsely negative NBS [22,25,26]. This occurred in four of our missed cases. For two additional missed cases with meconium ileus, IRT was appropriately elevated; however, no variants were detected on commonly used *CFTR* panels at the time. These findings support a recommendation for full *CFTR* gene sequencing for all infants with intestinal blockage due to meconium ileus, since timely sweat testing often cannot be obtained in the neonatal intensive care unit and NBS may miss some rare *CFTR* variants [27]. Like some states, Georgia added a meconium ileus checkbox to state NBS cards in 2024 so that second-tier *CFTR* testing can be performed for those samples regardless of IRT levels. The CF center directors have also conducted educational outreach to neonatal providers in the state regarding the recommendation for a full CF evaluation for infants with meconium ileus, even if the NBS is negative.

The CF Foundation recommends that infants with a positive NBS for CF undergo sweat chloride testing by four weeks of age to optimize early interventions [7]. Martiniano et al. have quantified the impact of delays in diagnosis on outcomes and found that infants who were diagnosed beyond this neonatal period had growth deficits that persisted into early childhood [10]. Though reasons for delay were not specifically studied, they showed that infants with a delay in diagnosis were more likely to have only one *CFTR* variant identified on commercially available NBS panels [10]. Along with poorer health outcomes, increased parental anxiety and medical mistrust have been shown among those with a child with a late CF diagnosis [28]. Often, reasons for delay in CF diagnosis are multi-factorial and include biases about infant ancestry, provider lack of state-specific NBS algorithm knowledge, and logistical barriers to confirmatory sweat testing. We found that 50% of delays in diagnosis for our CF patients with a positive NBS were related to sweat testing, either with difficulty scheduling and obtaining a sweat test or with inconclusive results. As expected, this occurred most commonly in infants with only one *CFTR* variant detected by NBS, supporting that expansion to larger genetic panels for NBS would not only impact the false negative rates but also mitigate delays in diagnosis by resulting in higher two-variant detection. Importantly, we identify a few children with missed or delayed diagnosis who carry at least one variant not listed in CFTR2 and could be missed if sweat testing were eliminated for those with only a single variant identified on an assay of all disease-causing variants in CFTR2, as considered in new guidelines [20].

Based on our learnings from our delayed cases and the identification of sweat testing as a common source of delay in diagnosis in our state, we initiated a statewide quality improvement project to improve sweat testing timeliness for infants with a positive NBS and only one-variant identified. We now track monthly average time to sweat testing completion and have implemented various interventions to improve processes, including sweat test ordering and scheduling. We also developed a pathway for the care centers to assist the Follow-up Program with challenging delays, for example, to help a family navigate transportation or insurance barriers. Additionally, we have updated the one-variant infant notification letter for primary care providers to better convey the urgency of sweat testing for infants of all races and ethnicities. We have also developed a parent-facing educational handout about sweat testing for one-variant positive infants. Our CF center directors are conducting educational outreach to providers around the state, including via a collaboration with the Georgia Chapter of the American Academy of Pediatrics. Key messages for primary care providers supported by our cases include the importance of prompt evaluation for infants with positive NBS, as well as for infants with a negative NBS and CF-type symptoms.

Because many of our missed cases were due to an IRT below the cut-off, we investigated alternative IRT approaches. The Association of Public Health Laboratories states that a floating IRT cut-off may be used to account for seasonal variation and kit-to-kit variability of the reagents [29], and a floating cut-off is now recommended by guidelines [20]. This recommendation was supported by examination of IRT data from Wisconsin, which showed large differences in IRT values in winter and summer months, suspected to be related to temperature sensitivity [26]. Fixed IRT cut-offs are often set at the 95–99th percentile; however, over time, IRT trends may change where fixed cut-offs are no longer at their intended thresholds [22]. Our data from Georgia, which is exposed to extremely warm temperatures, revealed the same patterns. We also found similar variation among reagent kits in Georgia as demonstrated in Wisconsin [26]. This means that the current use of a fixed cut-off may result in some infants having a lower chance of a positive screen based on birth season or reagent kit used and supports use of a floating or hybrid cutoff approach. Nevertheless, all our infants born between 2018 and 2022 with low IRT would have still been missed using the alternative cut-off approaches we explored. Low IRT false negatives may be due to patient characteristics or issues with sample collection, transport, or delays in testing [30]. Our state is currently conducting a project to improve dried blood spot specimen quality including educational outreach and feedback to state hospitals. More research is needed to explore how to further reduce false negatives due to low IRT, since these infants will be missed regardless of an algorithm’s *CFTR* variant expansion.

In addition to these improvement efforts, the Georgia Newborn Screening Advisory Committee has voted in favor of implementing a floating IRT cut-off and an expanded *CFTR* assay for CF NBS in the state. Evaluation of the feasibility and processes required for implementation is in process at the Department of Public Health. When considering expanded NGS-based *CFTR* assays for NBS, it is important to consider the higher costs and additional training that may be required to implement this approach.

Our study has several limitations. First, our true missed case rate is likely higher as some children with CF have not yet come to clinical attention. These individuals with missed diagnoses may be more likely to be of minoritized backgrounds. In addition, some individuals may have been diagnosed with CF after relocating to other states. Second, missed and delayed cases were not consistently tracked prospectively in the state. Thus, we may have had under-detection of cases, especially from the early years of NBS due to more limited data in the EMR from that time period and less historical memory from clinicians. Additionally, we only collected data on delayed cases since 2011, after the IRT cut-off last changed, due to limited accessible medical records to confirm delayed diagnosis and identify reasons for delay. We limited our OR analysis to this time because of misclassification that would occur for delayed cases prior to 2011. Importantly though, a sensitivity analysis, including all CF births since 2007, was consistent with the primary analysis. Third, we collected reasons for delayed diagnosis >28 days from retrospective chart review, and some reasons for delay, particularly social determinants of health or insurance status, may not have been well documented. Fourth, the population in Georgia as well as the CF population nationally is growing increasingly diverse [1,14], thus our historical data may underestimate false negative cases and delayed diagnoses occurring at present. Fifth, there are inherent limitations to the race/ethnicity data recorded by the nurse on the NBS card, which may result in misclassification. Lastly, our dataset did not allow further exploration of reasons for low IRT. Despite these limitations, our results reveal important opportunities for improving the CF newborn screening algorithm in Georgia.

## 5. Conclusions

The current CF NBS algorithm in Georgia has led to early detection of most infants with CF; however, minoritized CwCF have higher odds of missed or delayed diagnosis compared to non-Hispanic White children. Delayed diagnosis can contribute to disparities in long-term CF health outcomes. Employing an expanded *CFTR* panel in the NBS algorithm, addressing delays in sweat testing, and improving provider education on limitations of NBS would improve timely diagnosis and health equity in Georgia.

## Figures and Tables

**Table 1:** Demographics of children with cystic fibrosis born in Georgia from 2007 to 2022.

Characteristic	Overall	Missed	Missed (Low IRT)	Missed (Genetics)	Delayed
Total *n*	390	18	12	6	30
**Race**					
Black	37 (9%)	3 (17%)	3 (25%)	1 (17%)	6 (20%)
Multiracial	10 (3%)	1 (6%)	0 (0%)	0 (0%)	0 (0%)
Other race	9 (2%)	1 (6%)	0 (0%)	1 (17%)	2 (7%)
White	334 (86%)	13 (72%)	9 (75%)	4 (67%)	22 (73%)
**Ethnicity**					
Hispanic	27 (7%)	4 (22%)	0 (0%)	4 (67%)	6 (20%)
Non-Hispanic	363 (93%)	14 (78%)	12 (100%)	2 (33%)	24 (80%)
**Sex**					
Female	197 (51%)	8 (44%)	4 (33%)	4 (67%)	18 (60%)
Male	193 (49%)	10 (56%)	8 (67%)	2 (33%)	12 (40%)

Missed refers to a diagnosis of CF after a false negative NBS, which could be due to IRT below the state cut-off of 55 ng/mL or due to no *CFTR* variants detected. Delayed is a diagnosis of CF at >28 days of age despite positive NBS. Delayed cases were only collected from 2011 (after the state’s most recent IRT cut-off change) through 2022. Other race includes Other race, Asian, and American Indian/Alaskan Native. No patients identified as Pacific Islander/Native Hawaiian. Abbreviations: IRT, immunoreactive trypsinogen; NBS, newborn screening.

**Table 2:** Odds ratios for likelihood of having delayed and/or missed vs. on-time diagnosis for cystic fibrosis patients born in Georgia since 2011, according to race and ethnicity (*n* = 269).

Characteristic	Delayed and Missed vs. On-Time Diagnosis, *n* = 269		Missed Only vs. On-Time Diagnosis, *n* = 239		Delayed Only vs. On-Time Diagnosis, *n* = 254	
	OR (95% CI)	*p*-Value	OR (95% CI)	*p*-Value	OR (95% CI)	*p*-Value
**Race**						
Black	2.7 (1.1, 6.2)	**0.027**	2.7 (0.57, 9.4)	0.156	2.7 (0.90, 7.1)	0.057
Other Race	2.2 (0.47, 8.2)	0.254	2.2 (0.11, 14)	0.469	2.2 (0.32, 9.6)	0.330
White	—		—		—	
**Ethnicity**						
Hispanic	6.1 (2.4, 16)	**<0.001**	7.8 (1.9, 28)	**0.002**	5.3 (1.7, 16)	**0.003**
Non-Hispanic	—		—		—	

White race and Non-Hispanic ethnicity were used as reference groups to examine disparities. Due to small sample sizes, Other race includes Other race, Multiracial, Asian, and American Indian/Alaskan Native. No patients identified as Pacific Islander/Native Hawaiian. Abbreviations: CI = Confidence Interval, OR = Odds Ratio.

## Data Availability

Aggregated data available by reasonable request to the corresponding author.

## Supplementary Materials

The following supporting information can be downloaded at: https://www.mdpi.com/article/10.3390/ijns11040087/s1, Figure S1: Georgia’s Newborn Screening Follow-up Program algorithm for positive cystic fibrosis screens; Table S1: Cystic fibrosis cases missed by Georgia newborn screening from 2007–2022; Table S2: Days to cystic fibrosis diagnosis for delayed and missed cases, stratified by race and ethnicity; Table S3: Sensitivity analysis for the likelihood of having delayed and/or missed vs. on-time diagnosis for cystic fibrosis patients born in Georgia since 2007, according to race and ethnicity; Table S4: Historical immunoreactive trypsinogen (IRT) trends and comparison of cut-off algorithms; Table S5: Case detection for *CFTR* assays among children with cystic fibrosis missed by newborn screening in Georgia due to no variants detected

## Abbreviations

CF	Cystic fibrosis.
CFTR	Cystic Fibrosis Transmembrane Conductance Regulator.
CFTR2	Clinical and Functional Translation of CFTR.
CT	computed tomography.
CwCF	Children with cystic fibrosis.
CFFPR	Cystic Fibrosis Foundation Patient Registry.
DPH	Department of Public Health.
GPHL	Georgia Public Health Laboratory.
EMR	Electronic medical record.
NBS	Newborn screening.
IRT	Immunoreactive trypsinogen.
PwCF	People with CF.
U.S.	United States.
