# Supplementary material for: Evaluating Georgia’s Cystic Fibrosis Newborn Screening Algorithm to Inform Improvement Recommendations
Source: Int J Neonatal Screen. 2025 Sep 29;11(4):87. doi: 10.3390/ijns11040087 (PMC12551103; doi:10.3390/ijns11040087)
Supplement: Supplementary file 1 [file IJNS-11-00087-s001.zip › IJNS-3757444-supplementary.pdf]

SUPPLEMENTARY MATERIAL FOR:

Evaluating Georgia's Cystic Fibrosis Newborn Screening Algorithm to Inform Improvement Recommendations

Brittany Truitt [1,2], Eileen Barr [2,3], Angela Wittenauer [3], Andrew Jergel [1], Shasha Bai [1], Rossana Sanchez Russo [3], Kathryn E. Oliver [1], Kathleen McKie [4], Rachel W. Linnemann [1,2*]

Affiliations

[1]Department of Pediatrics, Emory University, Atlanta, GA, 30322, USA

[2]Children's Healthcare of Atlanta, Atlanta, GA, 30329, USA

[3]Department of Human Genetics, Emory University, Atlanta, GA, 30322

[4]Department of Pediatrics, Augusta University, Augusta, GA, 30912

Correspondence: rachel.linnemann@emory.edu, +1-404-785-5437

LIST OF SUPPLEMENTARY TABLES

Figure S1. Georgia's Newborn Screening Follow-up Program algorithm for positive cystic fibrosis screens

Table S1. Cystic fibrosis cases missed by Georgia newborn screening from 2007-2022

Table S2. Days to cystic fibrosis diagnosis for delayed and missed cases, stratified by race and ethnicity

Table S3. Sensitivity analysis for the likelihood of having delayed and/or missed vs. on-time diagnosis for cystic fibrosis patients born in Georgia since 2007, according to race and ethnicity

Table S4. Historical immunoreactive trypsinogen (IRT) trends and comparison of cut-off algorithms

Table S5. Case detection for *CFTR* assays among children with cystic fibrosis missed by newborn screening in Georgia due to no variants detected

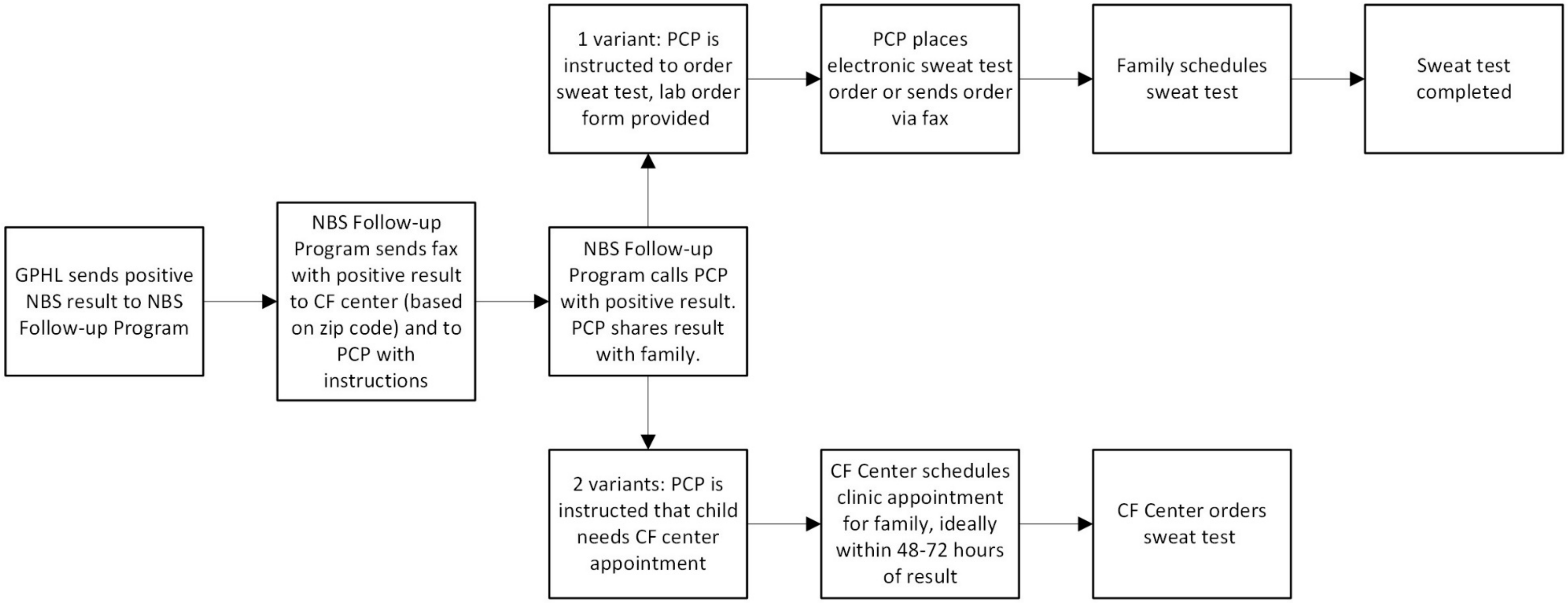

Figure S1. Georgia Newborn Screening (NBS) Follow-up Program algorithm for positive cystic fibrosis (CF) screens. After a positive NBS, the state's NBS Follow-up Program is responsible for coordinating the follow-up process until a final outcome is determined (case closure) and for tracking all outcome data. One-variant and two-variant results are reported by the NBS Follow-up Program to the PCP and to one of the state's two accredited CF Centers (Atlanta or Augusta, assigned based on child zip code). The PCP is responsible for informing all families of the NBS result. PCPs are instructed to immediately refer infants with two variants to the assigned CF Center for evaluation. For one variant infants, the PCP is responsible for ordering sweat testing from the assigned CF Center. NBS Follow-up Program staff follow up one week after the NBS result to ensure the sweat test has been ordered, scheduled and/or completed. Additional follow-ups to check for sweat testing results and communication with PCPs/families are determined on a case-by-case basis as needed to obtain a final diagnostic outcome. Once sweat testing is completed, PCPs are instructed to refer one-variant infants with abnormal sweat testing to the assigned CF Center for additional evaluation. If a one-variant infant has a normal sweat test, the follow-up program sends a carrier packet to the family and provider. The NBS Follow-up Program communicates with a CF center director for complex cases (recommendations for infants in a neonatal intensive care unit, prolonged delays in sweat testing, parent refuses sweat testing). After GPHL results the positive NBS, sweat testing and follow-up clinical care is paid by insurance or by families. GPHL: Georgia Public Health Laboratory; NBS: Newborn screening; PCP: primary care provider.

Table S1. Cystic fibrosis (CF) cases missed by Georgia newborn screening from 2007-2022

Case	Race	Ethnicity	IRT level (ng/mL)	Reason for being missed by NBS	Time from birth to CF diagnosis (days)	Symptoms at diagnosis	Sweat chloride (mmol/L)	CFTR variant analysis
**1**	Multiracial	Non-Hispanic	69.9†	IRT below cut-off	67	Meconium ileus	83, 71	F508del<br>CFTRdele2-4
**2**	White	Non-Hispanic	49.6	IRT below cut-off	777	Failure to thrive, cough	97, 90	F508del<br>R560K<br>Y569C*
**3**	White	Non-Hispanic	61.5†	IRT below cut-off	3127	Cough, sinusitis, younger sibling diagnosed with CF	53, 48‡	F508del<br>5T-TG13 (VVCC)
**4**	White	Non-Hispanic	37.6	IRT below cut-off	3539	Younger sibling diagnosed with CF	72, 73	F508del<br>2789+2insA (VVCC)
**5**	White	Non-Hispanic	47.3	IRT below cut-off	3571	Younger sibling diagnosed with CF	64, 68	F508del<br>2789+2insA (VVCC)
**6**	White	Non-Hispanic	40.9	IRT below cut-off	413	Diarrhea, malabsorption	88, 88	2789+5G>A<br>M1101K
**7**	White	Hispanic	246.4	No variants detected	2429	Malabsorption, diarrhea, older sibling diagnosed with CF	100, 101	1811+1634A->G<br>3271delGG
**8**	White	Non-Hispanic	60	No variants detected	187	Abnormal sweat test	58, 62	M1101K<br>Q1476X (VVCC)
**9**	Other	Hispanic	79.7	No variants detected	161	Meconium ileus	86, 85	2055del9->A<br>2055del9->A
**10**	White	Non-Hispanic	48.6	IRT below cut-off	65	Meconium ileus	94, 92	F508del<br>F508del
**11**	White	Non-Hispanic	29.8	IRT below cut-off	148	Cough, congestion, failure to thrive	99. 97	F508del<br>F508del
**12**	Black	Non-Hispanic	416.2	No variants detected	1530	Meconium ileus, failure to thrive	103, 105	CFTRdele1<br>CFTRdele21
**13**	White	Hispanic	73.1	No variants detected	81	Malabsorption, abdominal distention, failure to thrive	95, 99	1811+1G->A<br>1811+1G->A
**14**	Black	Non-Hispanic	42.3	IRT below cut-off	100	Abdominal distention, meconium plugs, cholestasis	84, QNS‡	3120+1G>A<br>Q552P*
**15**	Black	Non-Hispanic	46.7	IRT below cut-off	84	Malabsorption, older sibling diagnosed with CF	83, 83	F508del<br>3120+1G->A
**16**	White	Non-Hispanic	45.9	IRT below cut-off	24	Meconium ileus	94, QNS‡	F508del<br>F508del
**17**	White	Non-Hispanic	44.5	IRT below cut-off	694	Cough, frequent infections	74, 76‡	F508del<br>3120G->A
**18**	White	Hispanic	380.6	No variants detected	560	Cough, sinopulmonary infections, failure to thrive	102, 105	R1066C<br>W1204X

† Born prior to reduction in the IRT cut-off to 55 ng/mL in December 2010.

*Variant not listed in CFTR2 (September 2024 version).

‡Date of diagnosis differed from date of sweat test as diagnosis was made based on genetic testing ($n = 3$) or clinical presentation with a sibling previously diagnosed with CF (n=1). All other sweat test values correspond with date of diagnosis.

Abbreviations: IRT: immunoreactive trypsinogen; VVCC: Variant of varying clinical consequence per CFTR2; QNS: quantity not sufficient

Table S2. Age at cystic fibrosis diagnosis for delayed and missed cases, stratified by race and ethnicity

Characteristic	Age at diagnosis (days)[1]	p-value[2]
**Overall, *n* = 48**	75 (36, 180)	
**Race**		0.893
Black, *n* = 9	84 (33, 178)	
Other race[3], *n* = 4	92 (75, 116)	
White, *n* = 35	65 (36, 196)	
**Ethnicity**		0.732
Hispanic, *n* = 10	82 (50, 146)	
Non-Hispanic, *n* = 38	64 (35, 185)	
**Type of diagnosis**		**<0.001**
Delayed, *n* = 30	49 (33, 81)	
Missed, *n* = 18	300 (88, 1,342)	
**Variant detection on NBS for delayed cases, *n* = 30***		
1 Variant detected, *n* = 21	62 (30, 101)	**0.012**
2 Variants detected, *n* = 8	34 (31, 39)	

[1]Median (IQR)

[2]Wilcoxon rank sum test; Kruskal-Wallis rank sum test

[3]Due to small size, Other race category includes Multiracial and Other race.

*One infant with delayed diagnosis at 35 days had an inconclusive newborn screen on two occasions. The infant was therefore referred to the follow-up program and noted to have meconium ileus, so full sequencing was completed and resulted in a CF diagnosis.

Table S3. Sensitivity analysis for the likelihood of having delayed and/or missed vs. on-time diagnosis for cystic fibrosis patients born in Georgia since 2007, according to race and ethnicity, (*n* = 390)

**Characteristic**	**Delayed and Missed vs. On-Time Diagnosis, *n* = 390**		**Missed Only vs. On-Time Diagnosis, *n* = 360**		**Delayed Only vs. On-Time Diagnosis, *n* = 372**	
	**OR (95% CI)**	**p-value**	**OR (95% CI)**	**p-value**	**OR (95% CI)**	**p-value**
**Race**						
Black	2.7 (1.1, 6.1)	**0.017**	2.5 (0.54, 8.2)	0.178	2.9 (1.0, 7.4)	**0.033**
Other Race	2.3 (0.62, 6.7)	0.163	3.1 (0.45, 12)	0.164	1.8 (0.27, 7.0)	0.449
White	—		—		—	
**Ethnicity**						
Hispanic	5.0 (2.1, 12)	**<0.001**	5.5 (1.4, 17)	**0.006**	4.8 (1.6, 13)	**0.003**
Non-Hispanic	—		—		—	

White race and Non-Hispanic ethnicity were used as reference groups to examine disparities. Due to small sample sizes, Other race includes both Other race and Multiracial.
Abbreviations: CI = Confidence Interval; OR = Odds Ratio
Note: Delayed cases were not collected before 2011, so all non-missed diagnoses are assumed to be on-time in that time period, for the sensitivity analysis.

Table S4. Historical immunoreactive trypsinogen (IRT) trends and comparison of cut-off algorithms

**Characteristic**	**Overall** *n* = 715,639	**2018** *n* = 143,678	**2019** *n* = 144,428	**2020** *n* = 140,497	**2021** *n* = 142,974	**2022** *n* = 144,062
Characteristics of IRT, mean (SD)						
IRT (ng/mL)	27.13 (22.48)	27.17 (21.91)	26.70 (22.04)	26.65 (22.71)	27.34 (23.31)	27.77 (22.39)
96th Percentile	57.86 (4.42)	57.39 (4.16)	57.04 (4.23)	57.00 (4.41)	58.10 (4.23)	59.75 (4.47)
95th Percentile	54.34 (3.85)	53.96 (3.70)	53.65 (3.64)	53.45 (3.71)	54.55 (3.76)	56.06 (3.85)
Comparison of IRT cutoff approaches, n (column %)						
Fixed IRT at 55 ng/mL	37,407 (5%)	7,354 (5%)	7,181 (5%)	6,971 (5%)	7,549 (5%)	8,352 (6%)
Floating cutoff at daily 96th percentile	31,734 (4%)	6,340 (4%)	6,377 (4%)	6,240 (4%)	6,388 (4%)	6,389 (4%)
Floating IRT cutoff at 96th percentile with backup fixed IRT 60 ng/mL	33,249 (5%)	6,597 (5%)	6,585 (5%)	6,439 (5%)	6,699 (5%)	6,929 (5%)
Floating IRT cutoff at 96th percentile with backup fixed IRT 55 ng/mL	38,608 (5%)	7,598 (5%)	7,482 (5%)	7,292 (5%)	7,778 (5%)	8,458 (6%)
Floating cutoff at daily 95th percentile	38,872 (5%)	7,781 (5%)	7,822 (5%)	7,644 (5%)	7,797 (5%)	7,828 (5%)
Floating IRT cutoff at 95th percentile with backup fixed IRT 60 ng/mL	39,189 (5%)	7,830 (5%)	7,864 (5%)	7,673 (5%)	7,857 (5%)	7,965 (6%)
Floating IRT cutoff at 95th percentile with backup fixed IRT 55 ng/mL	41,693 (6%)	8,289 (6%)	8,205 (6%)	8,002 (6%)	8,366 (6%)	8,831 (6%)

Using each proposed IRT cut-off, the number of NBS samples which would have screened positive based on fixed, floating, or hybrid approach was calculated.

Table S5. Case detection (detection of at least one variant) for *CFTR* assays among children with cystic fibrosis missed by newborn screening in Georgia due to no variants detected

**Assay number of *CFTR* variants detected**	**Total n = 6**	**Black, non-Hispanic n = 1**	**Hispanic n = 4**	**White, non-Hispanic n = 1**
Luminex-39	0 (0%)	0 (0%)	0 (0%)	0 (0%)
Illumina-139	4 (67%)	0 (0%)	3 (75%)	1 (100%)
Wisconsin-689	5 (83%)	0 (0%)	4 (100%)	1 (100%)
CFTR2-1085*	5 (83%)	0 (0%)	4 (100%)	1 (100%)

*Includes only variants routinely detectable by next generation sequencing (NGS) in newborn screening laboratories (the infant in illustrative case 3 has two large deletions which would not be detected by NGS).
